# Two Years of Cotton *(Gossypium hirsutum L.)* Data from the Georgia Coastal Plain, USA

**DOI:** 10.1038/s41597-024-03716-z

**Published:** 2024-09-27

**Authors:** Alisa W. Coffin, Michael H. Cosh, Kathryn Pisarello

**Affiliations:** 1 USDA-ARS, Southeast Watershed Research Laboratory, Tifton, GA 31793 USA; 2 USDA-ARS, Hydrology and Remote Sensing Laboratory, Belstville, MD 21032 USA

**Keywords:** Agroecology, Agriculture

## Abstract

The sustainable management of Earth’s complex ecosystems requires an abundance of field data to support long term stewardship. Remotely sensed satellite data provide crucial supplements to field measurements and are essential for deriving key operational products for monitoring Earth systems. However, to accurately calibrate and validate the models used to develop monitoring datasets, coincident field measurements are required. In 2018 and 2019, data related to cotton (*Gossypium hirsutum L*.) crops were collected from five fields in two farms located in Georgia, USA. Collections were timed to coincide with satellite overpasses to support the development of remote sensing-based crop and soil data products. Data collected include soil moisture, plant water content, above ground biomass, crop height, plant phenology, and field management practices (row direction, row spacing, and plant density). The datasets include 512 records collected in 2018 and 303 records collected in 2019. The data are archived in the National Agricultural Library Ag Data Commons repository and are available for use by researchers seeking crop and soil validation data.

## Background & Summary

Effective management of Earth’s complex systems requires an abundance of data to support long term stewardship. However, data are not always ubiquitous across space and time, as data collection can be challenged by a lack of physical access, economic means, or a combination of both. Remotely sensed satellite data provide a crucial supplement to on-the-ground measurements and are therefore essential for monitoring Earth’s surface in near real time^1^. These data are routinely collected by passive and active sensors on orbital platforms and are made publicly available for scientific research and management applications. Importantly, some remotely sensed data are used to derive key operational products for monitoring Earth systems, such as processes that contribute to our understanding of the status of available food and water resources. For example, soil moisture is monitored globally by the Soil Moisture Active Passive (SMAP) mission^2,3^. The increasing availability of Earth observations at fine spatial and temporal resolutions offers opportunities for the improvement of models, tools, and operational data products that support decisions about the management of cropping systems, soils, and water resources^4,5^. However, to accurately calibrate and validate these models, key variables require ground truthing^6^. Intensive field campaigns that provide harmonized datasets, alongside well-designed procedures and guidelines, are needed to support global monitoring efforts^7–9^.

The United States Department of Agriculture – Agricultural Research Service (USDA-ARS) Southeast Watershed Research Laboratory (SEWRL) in Tifton, Georgia, United States of America (USA), has been engaged with several multidecadal efforts to contribute to the development of physical and statistical models informed by remote sensing techniques^6,10^. These efforts contribute to research collaborations including the USDA Conservation Effects Assessment Project (CEAP) Watershed Assessment Study (WAS) https://www.ars.usda.gov/anrds/ceap/ceap-home/, the USDA Long-Term Agroecosystem Research (LTAR) Network^11,12^, and the Joint Experiment for Crop Assessment and Monitoring (JECAM; http://jecam.org/documents/).

**Fig. 1:**
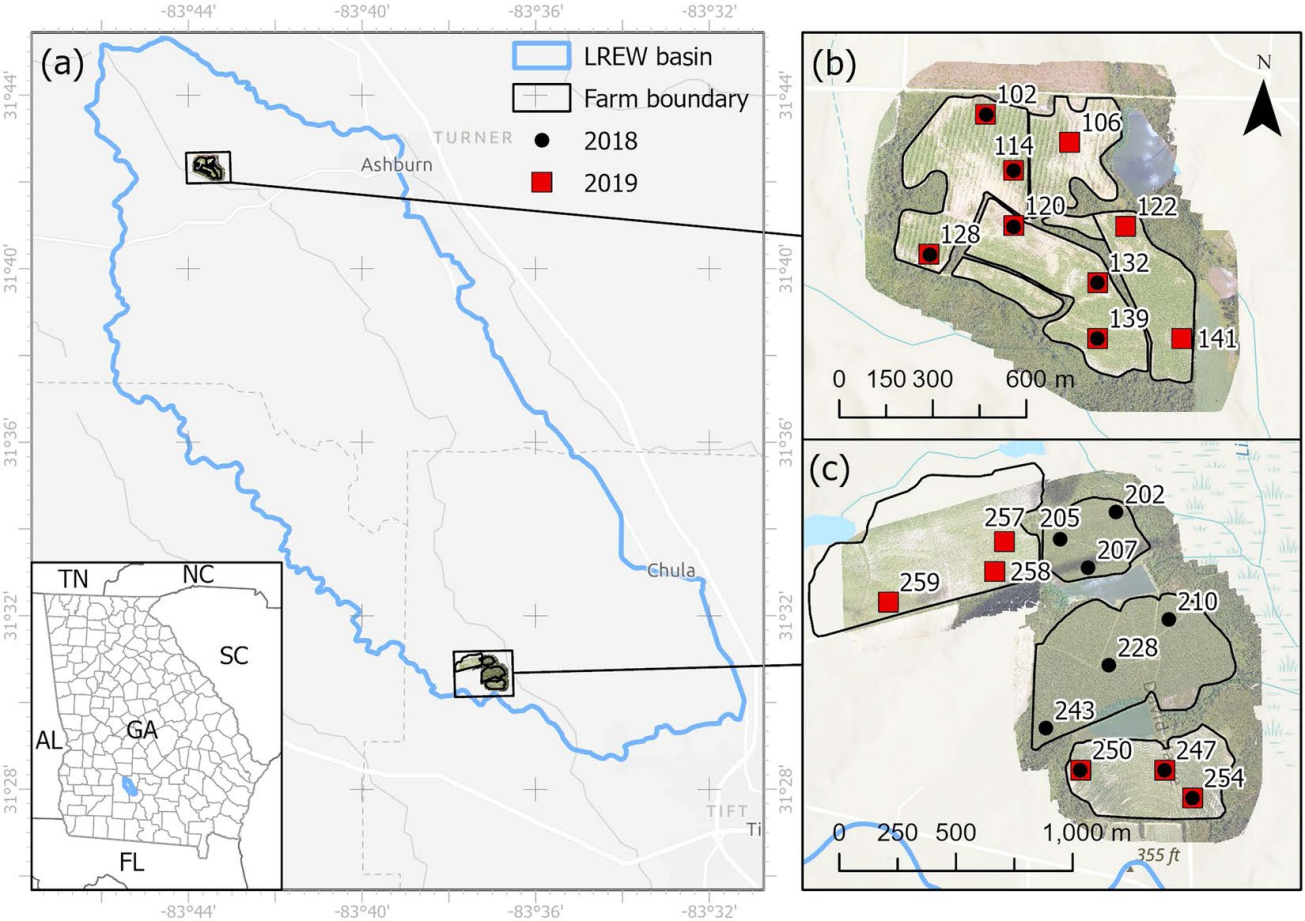
Map showing the location of the Little River Experimental Watershed (LREW) near Tifton, Georgia, USA, (**a**), and the location of the farms where field data were collected: Ashburn Cooperator Farm (**b**), and Ty Ty Cooperator Farm (**c**). Legend: In (**b**) and (**c**), black dots show sample sites in 2018, red squares show sample sites in 2019. Numbered labels correspond to the Idkey values for each site.

The SEWRL manages the Gulf Atlantic Coastal Plain LTAR Network site, which includes the Little River Experimental Watershed (LREW)^13,14^ (Fig. 1). The LREW is an ARS benchmark research watershed instrumented with weather and streamflow monitoring sites since the late 1960s. Rainfall, weather and soil moisture data from the monitoring network are publicly available through the data portal developed by CEAP-WAS, Sustaining the Earth’s Watersheds, Agricultural Research Data System (STEWARDS) v4.0^15^.

In the southeastern USA, where the LREW is located, frequent cloud cover can obscure the ground from optical sensors, limiting within season crop monitoring. These temporal measurement gaps hinder the efficacy of models to capture growing season dynamics and associated land management and climate impacts. Comprehensive agronomic planning strategies are therefore restricted. Synthetic aperture radar (SAR) is able to overcome challenges in cloud cover, versus optical and infrared remote sensing, because clouds do not obstruct radar signals. For this reason, SAR has the potential to add significantly to the operational measurement of regional cropping systems^16,17^.

To advance the use of SAR for crop mapping, in 2018 and 2019, the SEWRL participated in the Synthetic Aperture Radar Inter-Comparison Experiment, a multi-partner research effort facilitated by JECAM partners at Agriculture and Agrifood Canada (AAFC) (http://jecam.org/documents/)^18,19^. In previous work, Hosseini and McNairn^16^ demonstrated the use of multi-polarization C- and L-band SAR for biomass estimation of wheat using a Water Cloud Model (WCM). Cotton (*Gossypium hirsutum* L.) is a key commodity in the LREW region, and a calibrated WCM for cotton could improve remotely sensed estimates of cotton yield. In 2018 and 2019, data related to a cotton crops were collected from five fields across two farms in the LREW to provide calibration data toward this and other ongoing scientific collaborations (Table 1). Collections were timed to coincide with satellite overpasses from the Radarsat-2 L-band SAR and the Sentinel-1 C-Band SAR instruments (Supplementary Table 1). Data collected included soil moisture, plant water content (derived from above ground biomass), crop height, plant density, plant phenology, and field management practices (row direction, row spacing, key dates, and treatments). While these data contributed to the JECAM SAR Intercomparison Experiment, they may also prove valid for other research questions, such as those related to scaling above-ground biomass measurements, and evaluating the within-field variability of growing conditions.

**Table 1:** Summary of field activities.

Farm	Year	Plant_DOY	Defol_DOY	Harvest_DOY	SiteN	Start_DOY	End_DOY	RecordN	Dataset Name
ACF	2018	145	276	327	6	150	290	209	ACF_2018_f
TCF	2018	142	288	303	9	151	295	303	TCF_2018_f
ACF	2019	135	287	300	9	116	296	192	ACF_2019_f
TCF	2019	133	269 and 278	291 and 308	6	116	284	111	TCF_2019_f

Legend: Plant_DOY = day of year when planting occurred; Defol_DOY = day of year when defoliant was applied; Harvest_DOY = day of year when harvest occurred; SiteN = number of sites sampled; Start_DOY = day of year when sampling commenced; End_DOY = day of year when sampling ended; RecordN = total number of records in the dataset; ACF = Ashburn Cooperator Farm; TCF = Ty Ty Cooperator Farm.

## Methods

### Field sites and collection campaigns

Climate in the study region is humid and subtropical, with average precipitation rates of 1200 mm per year. Rainfall is distributed throughout the year, though July through October tend to be drier months. Summer months in the LREW basin experience convective weather patterns, with daily thunderstorms and tropical weather systems a common occurrence.

Two farms within the LREW basin were used for sample collections in 2018 and 2019: the non-irrigated Asbhurn Cooperator Farm (ACF) located near Asbhurn, Georgia (31° 42′24″ N, 83° 43′35″ W) at 125 meters above sea level (masl), and the irrigated Ty Ty Cooperator Farm (TCF) located near Ty Ty, Georgia (31° 30′41″ N, 83° 37′00″ W) at 100 masl (Fig. 1). Access for USDA-ARS research was provided by farm owners under a Cooperative Agreement. In accordance with prevailing regional production practices, crops in the fields at the farms followed shifting patterns of cultivation^20^, where cotton and peanuts comprise the major commodity crops grown in rotation. In all fields the cotton was planted in a common row spacing of approximately 91 cm. Planting dates for cotton were 18–25 May (DOY 138–145) in 2018, and 13–17 May (DOY 133–137) in 2019. Harvest dates, however, varied more widely. In 2019, for example, one cotton field at TCF was harvested over two weeks earlier than another field, possibly due to an accidental herbicide drift in the second week of June that affected the field where sites 257, 258, and 259 were located.

Management of the farms was carried out entirely by cooperators without the involvement of researchers. Planting and harvest dates along with management practices were observed and recorded for both sites. While irrigation was not measured, daily precipitation values during the study period are available through the STEWARDS data portal. For TCF and ACF, the most proximal STEWARDS rain gauge sites are GALR0012 and GALR0038 respectively.

Sites selected for sampling (i.e. sample sites) followed the guidelines described for the JECAM experiment^8^. During each year, three sample sites were established within fields planted to cotton, such that the fields and collection sites were sufficiently distant from field edges and from each other (60 m). Only fields with sufficient area to meet these distance criteria were used for the study. This distance allowed for field samples to appear at locations that, within a satellite image, would be surrounded by pixels of reflectance values associated with the plants and soils of the crop field itself and not with adjacent non-crop vegetation. Locations of the sample sites were selected from a pre-designed grid of points used by SEWRL researchers for long-term research to sample soils and crops. Soil conditions at all of the sample sites were similar, consisting of loamy sand soils (Tifton loamy sand, or Fuquay loamy sand) with 0 to 5 percent slopes.

For each year there are data for fifteen sites (Fig. 1(b,c)). However, since cotton was not planted consecutively in all fields in both years, data were collected at nine common sites across both years: three sites at TCF (247, 250, 254), and six sites at ACF (102, 114, 120, 128, 132, 139) were sampled in both 2018 and 2019. In 2018 there were six TCF sites (202, 205, 207, 210, 228, 235, 243) sampled only in 2018. In 2019, there were three ACF sites (106, 122, 141) and three TCF sites (257, 258, 259) that were sampled only in 2019.

Each field campaign in 2018 and 2019 involved sorties to collect samples and field data at both farms during the days (+/− 2 days) coinciding with an overpass from the Radarsat-2 (1138 UTC, -5 h for local standard time) and Sentinel-1 satellites (Supplementary Table 1). This resulted in a total of nine sorties per year between 30 May (DOY 150) and 17 Oct (DOY 290) in 2018, and between 26 Apr (DOY 116) and 23 Oct (DOY 296) in 2019, for a total of 18 data collections.

#### Sample site design and collection plan

**Fig. 2:**
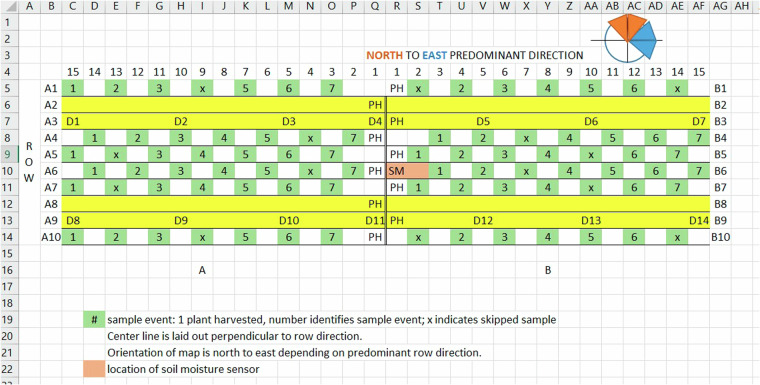
Sample site map showing data collection plan. Numbers across the top indicate meter length subsections (15-1, 1-15) of the sample site. Legend: **A**, left side of sample site; **B**, right side of sample site; SM, soil moisture measurement location (orange); PH, plant height measurement location; D1 through D14 were internal flag locations; yellow colored rows indicate areas where no destructive sampling occurred; green numbered blocks indicate biomass sampling locations by sortie number (x indicates no sample).

Fifteen sample sites were designed as 30 m × 10 row areas. Early in the growing season, sample sites were organized around a center point where either *in situ* soil moisture sensors or central marker flags (at five locations in 2018) were placed for the duration of the growing season. The soil moisture sensors were installed as close as possible to the selected location while ensuring they would not obstruct farming equipment. A Trimble Geo7X Global Navigation Satellite System receiver with real-time kinematic corrections from the VRS-Now system was used to record the precise locations of the center point, which was then designated as row number six in the sample site map (Fig. 2). Rows were then numbered perpendicularly as one through ten, with row one the most northerly or easterly. This created a a short central axis in the center of the sample site with two sides: A (left or south/west side), and B (right or north/east side). Lengths of 15 m were then measured off on both sides of this central axis using a nylon coated fiberglass 50 m measuring tape. Corners of the sample sites were flagged with 2 m tall brightly colored bicycle flags for ease of re-location, and small brightly colored flags were located along the rows at 5 m, 10 m, and 15 m distances from the center point to assist with orientation during sampling procedures.

With sample sites thus mapped, a collection plan was implemented to minimize the negative effects of destructive plant sampling on farming operations. Rows were segmented into 1 m sections and the sections were labeled 1 through 15 along the length of the rows from the center point to the far edges on each side. The specific 1 m sections pre-selected for destructive biomass sampling during each sortie were distributed throughout the sample sites as indicated by a green colored label on the map shown in Fig. 2. For example: in field sortie 1, plants were cut from rows 1, 4–7, and 10 at sections 14 and 15 on side A, and from rows 4–7 at Sections 2 and 3 on side B. The selected section shifted during the season according to the collection plan. The result was that no section was sampled more than once during the season. Four rows (2, 3, 8, and 9) were were protected from destructive sampling at each sample site.

#### Field collection procedure

At initiation of the field campaigns, measurements of row spacing, direction, and plant count were recorded. Throughout the field campaigns, basic management observations describing chemical and physical treatments were noted. Thereafter, field sorties included the collection of soil moisture data, plant samples, photographs, and measurements of plant height. Measurements and notes were written into bound field notebooks with pre-printed data collection sheets and were transcribed into a spreadsheet after returning to the lab. In 2019 soil moisture sensors were set out at seven sites (102, 106, 114, 128, 132, 247, and 254) prior to planting in order to test equipment and capture early season conditions. They were removed after 26 Apr (DOY 116) and reestablished immediately after planting at the same locations for collection during the 20 May (DOY 140) satellite overpass (Supplementary Table 1). A common practice for cotton cultivation is to apply plant growth regulators late in the season, followed by a defoliant about one month prior to harvest. The field campaigns continued throughout this period and ended 1 to 2 weeks after defoliant was applied, and prior to harvest.

### Soil moisture

Soil moisture and soil temperature data were collected using Stevens Water HydraProbe^®^ sensors. These sensors are a dielectric sensor which then uses a soil texture based algorithm (“sand” in this case) for conversion to a soil moisture estimate^21^. Soil moisture (i.e., soil water content) was measured in the units of *m*^3^/*m*^3^ and is defined as the volume of water per volume of soil. Soil temperature was recorded in degrees Celsius. A more detailed laboratory analysis of soil texture is underway, and intended for future publication.

**Fig. 3:**
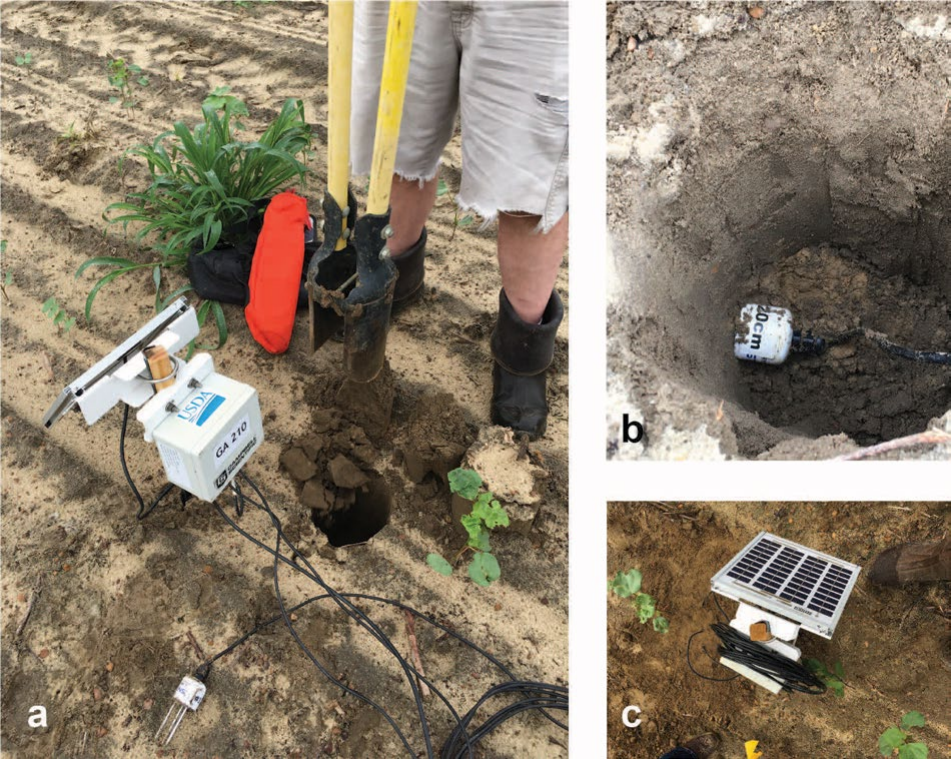
Photographs showing installation of soil moisture sensor (**a**), placement of sensor at 20 cm depth (**b**), and final installed *in situ* sensor (**c**).

The soil moisture instruments were deployed as shown in Fig. 1, with 10 units installed in 2018 and 15 units in 2019. Each unit included three probes that were installed with the central tine located at depths of 2.5 cm, 5 cm, and 20 cm (Fig. 3). Data were recorded every 60 minutes and formatted using routines in Campbell CR200X dataloggers powered by a battery charged with a solar photovoltaic panel. During each sortie, the data were exported as.csv files to a field computer.

After collection, soil moisture data were reviewed to ensure no obvious data gaps or errors. While the dataloggers ran continuously throughout the season, only data coinciding with the satellite overpass dates were selected for inclusion in the final dataset. The soil water content measurements for four time steps (0500, 0600, 0700, and 0800 local standard time) were selected for 2.5 cm and 5 cm depths. These values were averaged producing one soil water content value per site for 2.5–5 cm depth.

### Plant water content

Plant water content was derived from above ground biomass of the cotton. To calculate plant water content, 10 plants were harvested during each sortie according to the collection plan described above. Plant stems were clipped at the ground, and the entire plant was removed. After photographing the plants, they were combined into a single composite sample of 10 plants. The plants were placed into a bag and, within 3 hours, the composite samples were weighed in the laboratory using a tared Ohause Defender 5000 or a Mettler Toledo MS 3002S scale, and their values were noted as “wet weight”. After weighing, samples were placed in a drying oven at 60 °C for seven days until desiccated. After desiccation, samples were weighed again, with weights noted as “dry weight”. The resulting difference was recorded as grams of water lost (PWC). Averaged single plant weight was obtained by dividing this by 10 (*g*/*plant*). Single average plant weight was then multiplied by the plant density (*plants*/*m*^2^) to provide site level values of area weighted plant water content (PWC_g_m2). The percent plant dry matter content (PDC_PCT) was also calculated as the ratio of dry weight to wet weight × 100.

Plant density was evaluated after germination by counting the number of plants in 10 m sections of all rows in the sample site and averaging these values. Factoring with the average row distance, average plant counts were converted to a plant density value for each site. This was then used to calculate site level values of area weighted plant water content as noted above.

### Plant height and phenology

Plant height was measured in *cm*, where one plant per row was measured along the short central axis of each site. Plant height values were recorded during each field sortie and averaged (n = 10) to produce the average plant height per site.

**Fig. 4:**
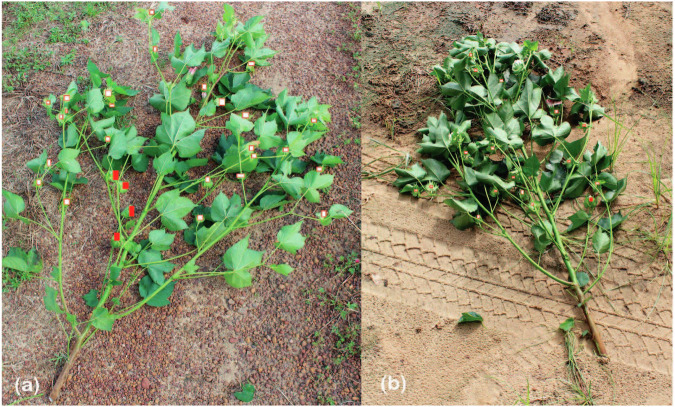
Photographs of cotton plants used for phenotyping using graphic markers to differentiate developing bolls (white boxes) from flowers (red boxes), from the Ty Ty Cooperator Farm (**a**), and from the Ashburn Cooperator Farm (**b**).

Phenology is the “study of the period of leafing, flowering, and fruiting in plants”^22^ and has been codified using the BBCH-scale. Munger *et al*.^23^ was used as a reference to evaluate and assign a two-digit BBCH-scale code to the phenological stage of the cotton plants collected in 2018. Photographs of plants collected from each site were closely evaluated (Fig. 4). Growth stage descriptions were compared with the photographic evidence, and a BBCH code was assigned to the overall site by a single evaluator. A second evaluator was consulted where a single code assignment was unclear, and the code was decided by consensus.

## Data Records

The data records include measurements of cotton plants and the fields where they were cultivated. Data records are comprised of plant water content (reported as grams, *g*/*m*^2^, and percent dry weight), crop height, crop phenology, and soil moisture. There are six data tables associated with the study. One table provides general information on planting density and row configuration for each year, one table provides all available management data for the farms for each year, and four tables provide detailed observations pertaining to two farms and two years of data. The 512 records from 2018 were collected from 25 May (DOY 150) to 22 Oct (DOY 295) (Table 2) and coincided with satellite overpasses on 12 dates in 2018 (Supplementary Table 1). In 2019, 303 records were collected from 26 Apr (DOY 116) to 23 Oct (DOY 296) (Table 3) and coincided with satellite overpasses on 9 dates in 2019 (Supplementary Table 1). Geospatial feature layers providing point locations of each sample site for each year were also created to facilitate spatial analysis. Each data record includes a unique identifier field (“Idkey”), which can be used to join to the geospatial data providing geographic coordinates of the sample location. Field characteristics were measured once at the start of the growing season and include plant spacing, row spacing, plant density, and planting direction. Plant characteristics include plant water content derived from values of wet and dry biomass and crop height. Management information includes the dates and types of treatment, and, where known, the trade names of chemicals applied.

**Table 2:** Field data collections in 2018, number of measurements.

DOY	Crop Height		Phenology		PWC- Biomass		Soil Moisture	
	ACF	TCF	ACF	TCF	ACF	TCF	ACF	TCF
150			6					
151				6				
156	6		6				4	
158		3		3				6
159		6		6				
169	6		6		6		4	5
170		9		9		9		
180	6	9	6	9			4	6
193	6		6		6		4	6
194		9		9		9		
205	6		6				4	6
206		9		9				
215	6		6		6			
218		9		9		9	4	6
229		9		9			4	6
241		1		1		1	4	5
242		8		8		8		
243	6		6		6			
253							4	5
263		9		9		9		
264	6		6		6		4	5
277	6		6				4	5
278		9		9				
288		2		2		2		
289							3	5
290	6		6		6			

Legend: DOY, day of year; ACF, Ashburn Cooperator Farm; TCF, Ty Ty Cooperator Farm.

**Table 3:** Field data collections in 2019, number of measurements (DOY, day of year; ACF, Ashburn Cooperator Farm; TCF, Ty Ty Cooperator Farm).

DOY	Crop Height		PWC- Biomass		Soil Moisture	
	ACF	TCF	ACF	TCF	ACF	TCF
116					5	2
140					5	2
164	7		8		7	5
165		6		6		
186	9		9			
188					9	6
189		6		6		
211		6		6		
212	9		9		9	6
235	9		9			
236					8	6
238		6		6		
260		6		6	8	6
261	9		9			
280		3		6		
283		3	9			
284	9				9	6
296	9		9		9	

Unexpected events and instrument failures affected data records for soil moisture and biomass leading to gaps in the records. In 2018, an instrument failure at site 228 in TCF before 29 Aug (DOY 241) resulting in a data gap for *in situ* soil moisture after that date. On 16 Oct (DOY 289) plant sample collections at TCF were limited to two sites due to a chemical application that prevented researchers from entering the field. Damage to sensor installations from wild animals occurred at both farms. This resulted in a data gap at ACF site 122 resulting in missing values for *in situ* soil moisture on 24 Aug 2019 (DOY 236) and 17 Sep 2019 (DOY 260).

Data records are archived in the USDA National Agricultural Library, Ag Data Commons repository and are publicly available^24^. Tabular data files are available as .xlsx files. Spatial data files are provided in.kml format, with a geographic projection using the WGS84 datum. In addition to the tabular joining field, latitude and longitude are also provided in the attributes of the spatial data.

## Technical Validation

### Soil moisture sensors

In this geography, previous studies have shown that the sand setting for the Stevens Hydra Probe has a good accuracy when compared to ground truth measurements^25,26^. For this study, all *in situ* sensors used the sand calibration equation in the setting for collection and calculation of soil water content.

### Plant characteristics data

Plant characteristics data were validated in several ways. Scales were calibrated annually and calibration information is available from the corresponding author. Data were checked twice, once when they were transcribed from field notebooks into datasheets, and again by a different person when the data were harmonized into a succinct dataset. Any errors that were discovered were corrected and checked again.

## Usage Notes

These data are ready for use with a geographic information system (GIS), where geospatial feature layers are provided along with the data tables in the repository. The join field (“Idkey”) can be used to join spatial data with the data tables in a one-to-many relationship. Software amenable to this analysis includes Esri ArcGIS Pro, and QGIS, among others. The spatial resolution of any subsequent analyses using these should be limited to an area no smaller than the area of the plots (30 m × 10 m) as a minimum mapping unit.

## Supplementary information

Supplementary Table 1

## Data Availability

No custom code was created for the production of this dataset.
