# Supplementary material for: Two Years of Cotton (Gossypium hirsutum L.) Data from the Georgia Coastal Plain, USA
Source: Sci Data. 2024 Sep 27;11:1037. doi: 10.1038/s41597-024-03716-z (PMC11437025; doi:10.1038/s41597-024-03716-z)
Supplement: Supplementary file 1 — Supplementary Table 1 [file 41597_2024_3716_MOESM1_ESM.docx]

Supplementary Table 1. Satellite and dates of satellite overpasses during 2018 and 2019 with data type collected. For crop height, biomass and phenology, field data collection may have occurred +/- 2 days from satellite collection date. Legend: CollectDOY = Day of year of satellite overpass; CollectDate = short date of satellite overpass (MM/DD/YYYY); Satellite = Satellite name; and DataType = soil moisture (SM), crop height (CH), BBCH phenology code (PH), and above ground biomass (B).

| Year | CollectDOY | CollectDate | Satellite | DataType |
| --- | --- | --- | --- | --- |
| 2018 | 157 | 6/6/2018 | Sentinel-1 | SM, CH, PH |
| 2018 | 169 | 6/18/2018 | Radarsat-2 | SM, CH, PH, B |
| 2018 | 181 | 6/30/2018 | Sentinel-1 | SM, CH, PH |
| 2018 | 193 | 7/12/2018 | Radarsat-2 | SM, CH, PH, B |
| 2018 | 205 | 7/24/2018 | Sentinel-1 | SM, CH, PH |
| 2018 | 218 | 8/6/2018 | Radarsat-2 | SM, CH, PH, B |
| 2018 | 229 | 8/17/2018 | Sentinel-1 | SM, CH, PH |
| 2018 | 241 | 8/29/2018 | Radarsat-2 | SM, CH, PH, B |
| 2018 | 241 | 8/29/2018 | Sentinel-1 | SM, CH, PH, B |
| 2018 | 253 | 9/10/2018 | Sentinel-1 | SM |
| 2018 | 264 | 9/21/2018 | Radarsat-2 | SM, CH, PH, B |
| 2018 | 277 | 10/4/2018 | Sentinel-1 | SM, CH, PH |
| 2018 | 289 | 10/16/2018 | Radarsat-2 | SM, CH, PH, B |
| 2018 | 289 | 10/16/2018 | Sentinel-1 | SM, CH, PH, B |
| 2019 | 116 | 4/26/2019 | Radarsat-2 | SM |
| 2019 | 116 | 4/26/2019 | Sentinel-1 | SM |
| 2019 | 140 | 5/20/2019 | Radarsat-2 | SM |
| 2019 | 140 | 5/20/2019 | Sentinel-1 | SM |
| 2019 | 164 | 6/13/2019 | Radarsat-2 | SM, CH, B |
| 2019 | 164 | 6/13/2019 | Sentinel-1 | SM, CH, B |
| 2019 | 188 | 7/7/2019 | Radarsat-2 | SM, CH, B |
| 2019 | 188 | 7/7/2019 | Sentinel-1 | SM, CH, B |
| 2019 | 212 | 7/31/2019 | Radarsat-2 | SM, CH, B |
| 2019 | 212 | 7/31/2019 | Sentinel-1 | SM, CH, B |
| 2019 | 236 | 8/24/2019 | Radarsat-2 | SM, CH, B |
| 2019 | 236 | 8/24/2019 | Sentinel-1 | SM, CH, B |
| 2019 | 260 | 9/17/2019 | Radarsat-2 | SM, CH, B |
| 2019 | 260 | 9/17/2019 | Sentinel-1 | SM, CH, B |
| 2019 | 284 | 10/11/2019 | Radarsat-2 | SM, CH, B |
| 2019 | 284 | 10/11/2019 | Sentinel-1 | SM, CH, B |
| 2019 | 296 | 10/23/2019 | Sentinel-1 | SM, CH, B |
